# Adrenal lymphangioma removed by a retroperitoneoscopic procedure

**DOI:** 10.3892/ol.2012.1059

**Published:** 2012-12-04

**Authors:** BEN LIU, YANYUAN LI, SHUO WANG

**Affiliations:** 1 Departments of Urology, The First Affiliated Hospital, College of Medicine, Zhejiang University, Hangzhou, Zhejiang 310003, P.R. China; 2 Pathology, The First Affiliated Hospital, College of Medicine, Zhejiang University, Hangzhou, Zhejiang 310003, P.R. China

**Keywords:** lymphangioma, adrenal, retroperitoneoscopy

## Abstract

We report a case of an adrenal lymphangioma removed by retroperitoneal laparoscopy. A 45-year-old female was referred to the urological ward for an adrenal mass that was incidentally detected by ultrasound examination one month earlier. An abdominal ultrasonography (US) scan revealed a 3.0 cm anechoic cystic mass, while a computed tomography (CT) scan revealed a 3.0×2.7 cm left adrenal cystic mass, which was suspected to be an adrenal cyst. The patient underwent retroperitoneoscopic removal of the tumor. Pathological evaluation revealed a cystic lymphangioma in the left adrenal gland.

## Introduction

Adrenal lymphangiomas are rare and non-functioning benign tumors, which are usually asymptomatic. Laboratory findings are nonspecific. As imaging techniques have improved, Adrenal lymphangiomas usually appear as incidental findings at abdominal ultrasonography and computed tomography scan examinations. The most effective treatment option is surgical removal of the tumor. Herein, we report a case of an adrenal lymphangioma that was removed by retroperitoneal laparoscopy.

## Case report

**Figure 1.**
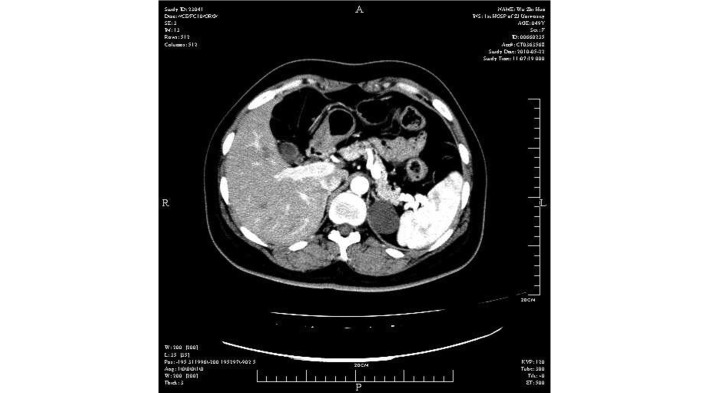
Contrast-enhanced computed tomography (CT) scan revealing a low density mass in the left adrenal gland.

A 45-year-old female was referred to the urological ward of The First Affiliated Hospital, College of Medicine, Zhejiang University, China, for an adrenal mass that had been detected incidentally by ultrasound examination one month earlier. The physical examination results were normal. A hormonal examination revealed that the function of the left adrenal gland was also normal. An abdominal ultrasonography (US) scan revealed a 3.0-cm anechoic cystic mass of the left adrenal gland. A computed tomography (CT) scan revealed a 3.0×2.7-cm hypodense non-enhancing lesion of the left adrenal gland, which was suspected to be an adrenal cyst (Fig. 1).

**Figure 2.**
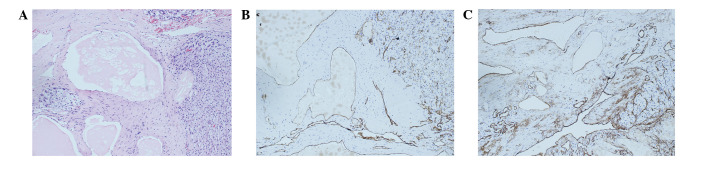
Histologically cystic lesion covered by flat endothelial cells adjacent to the adrenal gland that is normal in appearance (A). Flat endothelial cells are positive for CD31 (B) and CD34 (C).

Retroperitoneoscopic removal of the tumor was successfully performed via a posterior approach. For the first trocar insertion, a 2.0-cm transverse incision was made at the midaxillary line, approximately 3 finger-breadths cephalad to the *crista iliaca*. Two additional supracostal trocars (5 and 10 mm) were subsequently inserted in the anterior and posterior axillary lines, respectively. These two trocars were then guided by the index finger into the first port. Thereafter, a pneumoretroperitoneum was created by maintaining a carbon dioxide pressure of ∼5 mmHg, and retroperitoneoscopy was performed. Following confirmation of the location of the tumor, the tumor was successfully mobilized and removed using an ultrasonically activated scalpel and clip. The tumor was removed through an incision and subsequently placed in a specimen bag. The operating time was 80 min and blood loss was ∼30 ml. A drainage tube was put in place for 2 days. No complications were observed either intra- or postoperatively. No recurrence has been observed in the 6-month follow-up period. Pathological evaluation revealed a cystic lymphangioma in the left adrenal gland (Fig. 2).

## Discussion

Lymphangiomas are benign malformations of lymphatic vessels that originate from an abnormal embryologic development of the lymphatic system. There are four histological subtypes of lymphangiomas: cystic, capillary, cavernous and vasculolymphatic malformation (1). Combinations of such types may be seen in the same lesion. The dominating histological feature of lymphangiomas is endothelial-lined lymphatic channels, which are separated by connective tissue (2).

Lymphangiomas of the adrenal gland are rare. They are characterized by multiloculated cystic- and endothelial-lined cavities (3). The endothelial lining reacts with Factor VIII-related antigen, CD31 and CD34 (4). Adrenal lymphangiomas are typically asymptomatic; they are discovered as incidental findings in abdominal US and CT scan examinations, due to improved imaging techniques. Surgical removal of adrenal lymphangiomas is the most effective treatment. Retroperitoneoscopic removal of the tumor is regarded as a safe, effective and minimally invasive approach. This treatment option offers several advantages, such as an excellent visualization of the adrenal vessels allowing early ligation, and avoiding opening of the peritoneal cavity and manipulation of the intra-abdominal organs. Although the work space is limited for this procedure, retroperitoneal laparoscopy is usually performed without any difficulties. Therefore, it may become the standard treatment procedure for adrenal lymphangioma.
